# *Pseudoprevotella muciniphila* gen. nov., sp. nov., a mucin-degrading bacterium attached to the bovine rumen epithelium

**DOI:** 10.1371/journal.pone.0251791

**Published:** 2021-05-20

**Authors:** Sang Weon Na, Byung Hee Chun, Seok-Hyeon Beak, Shehzad Abid Khan, Md. Najmul Haque, Jae Sung Lee, Che Ok Jeon, Sang-Suk Lee, Myunggi Baik

**Affiliations:** 1 Department of Agricultural Biotechnology and Research Institute of Agriculture and Life Sciences, College of Agriculture and Life Sciences, Seoul National University, Seoul, Republic of Korea; 2 Department of Life Science, Chung-Ang University, Seoul, Republic of Korea; 3 Department of Animal Science & Technology, Sunchon National University, Sunchon, Republic of Korea; Babasaheb Bhimrao Ambedkar University, INDIA

## Abstract

A Gram-negative, strictly anaerobic mucin-degrading bacterium, which we designated strain E39^T^, was isolated from the rumen epithelium of Korean cattle. The cells were non-motile and had a coccus morphology. Growth of strain E39^T^ was observed at 30–45°C (optimum, 39°C), pH 6.5–8.5 (optimum, pH 7.5), and in the presence of 0.0–1.0% (w/v) NaCl (optimum, 0.0–0.5%). Strain E39^T^ contained C_16:0_, C_18:0_, C_18:1_ *ω*9*c*, iso-C_15:0_, and anteiso-C_15:0_ as the major fatty acids. The major polar lipids were phosphatidylethanolamine, unidentified aminophospholipid, and unidentified lipids. The major respiratory isoprenoid quinones were MK-8 and MK-9. The major fermented end-products of mucin were acetate and succinate. The G+C content of the genomic DNA was 46.4 mol%. Strain E39^T^ was most closely related to *Alloprevotella rava* 81/4-12^T^ with an 87.3% 16S rRNA gene sequence similarity. On the basis of phenotypic, chemotaxonomic, and molecular properties, strain E39^T^ represents a novel genus of the family *Prevotellaceae*; as such, the name *Pseudoprevotella muciniphila* gen. nov., sp. nov. is proposed. A functional annotation of the whole genome sequences of *P*. *muciniphila* E39^T^ revealed that this bacterium has a putative mucin-degrading pathway and biosynthetic pathways of extracellular polymeric substances and virulence factors which enable bacteria to adhere to the epithelial cells and avoid the host’s immune responses.

## Introduction

Rumen, a forestomach of ruminants, is a complex symbiotic ecosystem. In the rumen, various types of microorganisms (archaea, bacteria, protozoa, fungi, and virus) exist and they are known as the rumen microbiota. The rumen microbiota provides nutrients for the host ruminants by digestion of feed particles, especially indigestive fibrous material, and biosynthesis of metabolites like amino acids, lipids, and vitamins [1]. The nutrients including volatile fatty acids, minerals, and other metabolites are absorbed and transported into the host’s blood vessels through the rumen epithelium [2]. Only small portion of rumen microbiota attaches to the rumen epithelium. Several rumen epithelial bacteria (epimural bacteria) and their functions in urea digestion, tissue recycling, and oxygen scavenging have been identified since 1960s [3, 4]. Recent studies showed the composition and expressed genes of the epimural community in response to subacute rumen acidosis challenges using meta-omics technologies [5, 6]. Although these culture-independent methods provide expansive information about the epimural community, culture-dependent studies about yet uncultured eprimural bacteria are essential to understand their functions in more depth.

Mucin is a host-derived glycoprotein composed of protein backbones and various oligosaccharides. Intestinal epithelial cells produce two types of mucins (secreted mucin and cell membrane-associated mucin). They have a role as barriers to prevent pathogens from penetrating the epithelial tissues [7]. Mucin-degrading bacteria can degrade mucin and utilize it as an energy source. Some of them penetrate into the host epithelial cells with their mucin-degrading ability and show pathogenicity, others play roles as commensal bacteria [8]. Studies on *Akkermansia muciniphila*, a commensal mucin-degrading bacterium, revealed that this bacterium improved the host metabolic disorders, such as obesity and insulin resistance, by controlling gut barrier permeability and inflammation [9, 10].

In the bovine rumen, there are salivary mucin secreted from salivary glands and cell-membrane associated mucins expressed on rumen epithelial cells [11]. In the 1960s, Fina *et al*. isolated five uncultured mucin-degrading rumen bacteria from rumen fluid and Mishra *et al*. determined mucin-degrading activity of several species of rumen bacteria (*Butyrivibrio fibrisolvens*, *Selenomonas ruminantium*, *Streptococcus bovis*, *Peptostreptococcus elsdenii*, and *Bacteroides ruminicola*) [12–14]. They focused on mucin-degrading bacteria floating in the rumen fluid and their roles in bloat syndrome. Subsequently, there has been few studies on mucin-degrading bacteria floating in the rumen fluid nor them attached to the rumen epithelium.

In this study, we used mucin as a novel carbon source to enrich previously uncultured mucin-degrading bacteria from the rumen epithelium of Korean cattle. We isolated a novel mucin-degrading bacterium, called strain E39^T^. Then, we performed phylogenetic analysis, phenotypic and chemotaxonomic characterization, and genome analysis. On the basis of phenotypic, chemotaxonomic, and molecular properties, strain E39^T^ represents a novel genus of the family *Prevotellaceae*, for which we propose the designation *Pseudoprevotella muciniphila* gen. nov., sp. nov., strain E39^T^.

## Materials and methods

### Ethical statement

All experimental procedures were performed in accordance with the Animal Experimental Guidelines provided by the Seoul National University Institutional Animal Use and Care Committee, Republic of Korea. The experimental protocol was approved by the Seoul National University Institutional Animal Use and Care Committee (SNU-170626-1).

### Enrichment and isolation of mucin-degrading bacteria

Strain E39^T^ was isolated from the rumen epithelium of Korean cattle. Rumen epithelium tissue samples were excised from the ventral sacs of rumens immediately after slaughter at the abattoir in Bucheon (37°31’48.4"N 126°45’46.3"E), South Korea in 2017 and transported in a sterile container with rumen fluid. On arrival at the laboratory, the samples were moved into an anaerobic chamber and washed several times with anaerobic dilution solution (ADS; 3 g K_2_HPO_4_, 6 g NaCl, 3 g KH_2_PO_4_, 0.6 g CaCl_2_·2H_2_O, 0.6 g MgSO_4_·7H_2_O, 6 g (NH_4_)_2_SO_4_, 0.5 g cysteine-HCl, 0.5 g Na_2_S·9H_2_O, 0.625 g NaOH, and 1 mg resazurin per liter) to remove the rumen contents and non-adherent bacteria [15, 16]. Five grams of epithelial samples that were stripped from the muscle layer were homogenized in 30 ml of ADS and serially diluted (10-fold). Each 0.3 ml of dilution was inoculated into 30 ml of basal mucin medium in a butyl rubber stopped serum bottle and incubated at 39°C in an anaerobic atmosphere (95% CO_2_ 5% H_2_) for 24 h for enrichment. The basal mucin medium was prepared by modifying medium 10 [17] and consisted of 2 g peptone, 0.5 g yeast extract, 2.5 g hog gastric mucin (Type Ⅲ; Sigma), 50 ml mineral solution 1 (6 g K_2_HPO_4_ per liter), 50 ml mineral solution 2 (12 g NaCl, 6 g KH_2_PO_4_, 1.2 g CaCl_2_·2H_2_O, 1.2 g MgSO_4_·7H_2_O, and 12 g (NH_4_)_2_SO_4_ per liter), 10 ml Pfenning’s solution (0.5 g EDTA, 0.1 g ZnSO_4_·7H_2_O, 0.03 g MnCl_2_·4H_2_O, 0.03 g H_3_BO_3_, 0.2 g CoCl_2_·6H_2_O, 0.01 g CuCl_2_·2H_2_O, 1.5 g FeCl_2_·4H_2_O, 0.02 g NiCl_2_·6H_2_O, 0.03 g Na_2_MoO_4_·2H_2_O, and 0.01 g Na_2_SeO_3_ per liter), 10 ml volatile fatty acid solution (700 ml 0.2 N NaOH, 17 ml acetic acid, 6 ml propionic acid, 4 ml butyric acid, 1 ml iso-butyric acid, 1 ml 2-metylbutyric acid, 1 ml valeric acid, and 1 ml isovaleric acid, pH 7.5 per liter), 1 ml hemin solution (0.5 g hemin and 10 ml 1N NaOH per liter), 1 ml resazurin (0.1%, w/v), 20 ml cysteine sulfide solution (6.25 g NaOH, 25 g cysteine-HCl, and 25 g Na_2_S·9H_2_O per liter), and 4 g NaHCO_3_ per liter. The enrichments were serially diluted with ADS, streaked onto basal mucin agar medium, and incubated at 39°C for 4 days under anaerobic conditions. Each colony was picked, inoculated into 5 ml of basal mucin medium in a Hungate tube, and incubated at 39°C for 24–48 h. Streaking, colony picking, and incubation were repeated until an isolate was pure. The isolates were preserved with 15% (v/v) glycerol stock solution and stored at –80°C.

### Bacterial growth and genomic DNA extraction

Cells of strain E39^T^ were grown in 250 ml basal mucin medium at 39°C for 24 h and harvested by centrifugation (10,000 rpm for 5 min). The genomic DNA of the cells was extracted, according to standard procedures, including phenol-chloroform extraction and ethanol precipitation [18].

### 16S rRNA gene based phylogeny

The 16S rRNA gene of strain E39^T^ was amplified with PCR using the universal primers 10F (5′-AGT TTG ATC ATG GCT CAG ATT G-3′) and 1507R (5′-TAC CTT GTT ACG ACT TCA CCC CAG-3′) [19] and the resulting amplicons were sequenced using the universal primers 340F (5′-CCT ACG GGA GGC AGC AG-3′), 518R (5′-ATT ACC GCG GCT GCT GG-3′), and 805F (5′-GAT TAG ATA CCC TGG TAG TC-3′). The sequencing quality was checked, and the sequences were assembled using the Geneious program (ver. 11.0.4). The almost complete 16S rRNA gene sequence (1,476 nucleotides) of strain E39^T^ was compared with that of all validated type strains using the Nucleotide Similarity Search program in the EzBioCloud server (http://www.ezbiocloud.net/identify/) [20]. Phylogenetic trees were constructed with MEGA (ver. 7.0.26) using the neighbor-joining (NJ), maximum-parsimony (MP), and maximum-likelihood (ML) methods [21]. The complete 16S rRNA gene sequence (1,540 nucleotides) from the genome sequence of strain E39^T^ was used to construct the phylogenetic trees.

### Phenotypic and chemotaxonomic characterization

The cell morphology of cells grown on basal mucin agar medium at 39°C for 3 days was investigated using phase-contrast microscopy and transmission electron microscopy (Talos L120C; FEI) at 120 kV. Gram staining was performed using a Sigma Gram staining kit following the manufacturer’s protocol. The growth of strain E39^T^, as determined from the optical density (OD) at a wavelength of 600 nm, was evaluated by culturing the cells in basal mucin medium, brain heart infusion (BHI) broth (BD), trypticase soy broth (TSB; BD), Columbia broth (Acumedia), and anaerobe basal broth (Oxoid) at 39°C for 48 h. The optimum temperature, pH, and NaCl concentration for growth were determined by culturing the cells on basal mucin medium for 48 h at different temperatures (5–45°C, at 5°C intervals), pH (5.0–9.0 at 0.5 pH unit intervals), and NaCl concentrations (0.0–2.0% at 0.5% intervals). To determine the optimum pH, different pH buffers were used in the appropriate pH range (Na_2_HPO_4_-NaH_2_PO_4_ buffer at pH 5.0–7.5; Tris-HCl buffer at pH 8.0–9.0) and the pH values were adjusted before and after autoclaving (121°C, 15 min) [22]. Oxygen tolerance was investigated by measuring growth (OD at 600 nm) in the absence of a reducing agent (cysteine sulfide solution) or in the aerobic condition on basal mucin medium. *A*. *tannerae* ATCC 51259^T^, *A*. *rava* 81/4-12^T^, *Paraprevotella clara* YIT 11840^T^ and *Prevotella melaninogenica* ATCC 25845^T^, which is the type species of the genus *Prevotella*, were used as reference strains to compare enzyme profiles and cellular fatty acid composition. The enzyme profiles were determined using an API Rapid ID 32A identification kit (bioMérieux) following the manufacturer’s instructions. Analysis of cellular fatty acids was performed according to a standard MIDI protocol. All of the strains were cultivated in peptone-yeast extract-glucose (PYG) broth, except strain E39^T^, which was cultivated in basal mucin medium. Cells were harvested at the late exponential phase and cellular fatty acids were extracted from the cells following four steps (saponification, methylation, extraction, and base wash). Fatty acid methyl esters were analyzed by gas chromatography (Hewlett Packard 6890) and identified using the RTSBA6 database of the Microbial Identification System (Sherlock ver. 6.0B) [23]. The polar lipid profiles were analyzed by thin-layer chromatography following the Minnikin *et al*. method [24]. The following reagents were used to detect different types of polar lipids: 10% ethanolic molybdophosphoric acid (for total lipids), ninhydrin (for aminolipids), Dittmer-Lester reagent (for phospholipids), and α-naphthol (for glycolipids). The isoprenoid quinones of strain E39^T^, *A*. *tannerae* ATCC 51259^T^, *A*. *rava* 81/4-12^T^, *P*. *clara* YIT 11840^T^, and *P*. *melaninogenica* ATCC 25845^T^ were extracted from their exponentially grown cells according to the procedure described by Jeon *et al*. [25] and analyzed at 40°C using an Agilent infinity 1290 UHPLC equipped with a photodiode array detector (PAD) and an Agilent 6550 ifunnel Q-TOF MS (Agilent Technologies, USA). Briefly, 2 μl of quinone samples were injected into an Agilent Eclipse Plus C-18 column (2.1 mm × 100 mm, 2.1 μm) and eluted at 40°C using water (A) and acetonitrile (B) containing 0.1% formic acid as a mobile phases with the following gradient: 0 min, 85% B; 30 min, 100% B; 40 min; and flow rate, 0.4 ml/min. Isoprenoid quinone peaks in the chromatograms were identified by their UV spectra generated by PAD and their molecular masses were assessed using Q-TOF MS. The mass spectrometry was performed under the following conditions: polarity, positive; gas temp, 250°C; nebulizer, 35 psi; capillary, (+) 4,000 V; MS range, 100–1,500 m/z.

### Metabolite analysis using ^1^H NMR spectroscopy

Metabolic compounds including amino acids, monosaccharides, and organic acids in cultured broth of strain E39^T^ were analyzed using ^1^H NMR spectroscopy, as described previously [26]. Briefly, the basal mucin medium (2.5 g hog gastric mucin per liter; no glucose), glucose medium (5 g glucose per liter; no hog gastric mucin), and mucin-glucose medium (2.5 g hog gastric mucin and 5 g glucose per liter) were prepared based on the basal mucin medium to investigate mucin and glucose utilization by strain E39^T^ and their fermentation products. Strain E39^T^ was cultured in 5 ml of each broth at 39°C for 0, 9, 18, 27, 36, and 54 h. The growth of the cells was monitored by measuring OD at 600 nm. The culture broths were centrifuged, filtered with a 0.45 μm syringe filter, and 0.3 ml of filtrate was mixed with 0.3 ml of 99.9% D_2_O (Sigma-Aldrich, USA) containing 5 mM sodium 2,2-dimethyl-2-silapentane-5-sulfonate (DSS, 97%; Sigma-Aldrich). The mixtures were transferred into NMR tubes and their ^1^H NMR spectra were measured on Varian Inova 600-MHz NMR spectrometer (Varian, USA). Metabolic compounds were identified and quantified using the Chenomx NMR Suite program (ver. 6.1; Chenomx, Canada).

### Genome sequencing and analysis

De novo genome sequencing was performed using a Pacific Biosciences (PacBio) RSII platform at Macrogen (Seoul, Korea; http://www.macrogen.com). A library was prepared using PacBio DNA Template Prep Kit 1.0. After sequencing, reads were trimmed to obtain high quality region and then assembled using RS hierarchical genome assembly process (HGAP ver. 3.0) [27]. The complete genome was annotated using a software tool Prokka with default parameter (ver. 1.12) [28].

For the bacterial core genes-based phylogenetic analysis, 92 up-to-date bacterial core genes were extracted from the genomes of strains in the family *Prevotellaceae* and multiple-aligned, and a phylogenetic tree was constructed using the up-to-date bacterial core gene (UBCG) tool ver. 3 (https://www.ezbiocloud.net/tools/ubcg) [29]. The average nucleotide identity (ANI) and digital DNA-DNA hybridization (dDDH) values among the genomes of strain E39^T^ and reference strains were calculated using a stand-alone software (http://www.ezbiocloud.net/sw/oat) [30] and the Genome-to-Genome Distance Calculator (GGDC) ver. 2.1 (http://ggdc.dsmz.de/distcalc2.php) [31], respectively. Functional annotation of predicted proteins was performed using BlastKOALA tool of Kyoto Encyclopedia of Genes and Genomes (KEGG) (http://www.kegg.jp/blastkoala/) [32].

To predict putative mucin-degrading enzymes and carbohydrate active enzymes (CAZymes), the genome sequences of strain E39^T^ and reference strains were submitted to a meta server for automated carbohydrate-active enzyme annotation (dbCAN2) (http://cys.bios.niu.edu/dbCAN2/) [33]. VRprofile (http://bioinfo-mml.sjtu.edu.cn/VRprofile) was used for the prediction of virulence and antibiotic resistant genes [34].

### Nucleotide sequence accession number

The 16S rRNA gene and genome sequence of strain E39^T^ were deposited in GenBank under MG763147 and CP033459, respectively. The genome data of reference strains: *A*. *tannerae* ATCC 51259^T^ (GenBank acc. no., ACIJ00000000), *A*. *rava* 81/4-12^T^ (GenBank acc. no., ACZK00000000), *P*. *clara* YIT 11840^T^ (GenBank acc. no., AFFY00000000), *P*. *melaninogenica* ATCC 25845^T^ (GenBank acc. no., CP002122-3), and *Bacteroides thetaiotaomicron* VPI 5482^T^ (GenBank acc. No., AE015928) were obtained from GenBank for comparative analysis.

## Results and discussion

### 16S rRNA gene and genome based phylogeny

**Fig 1:**
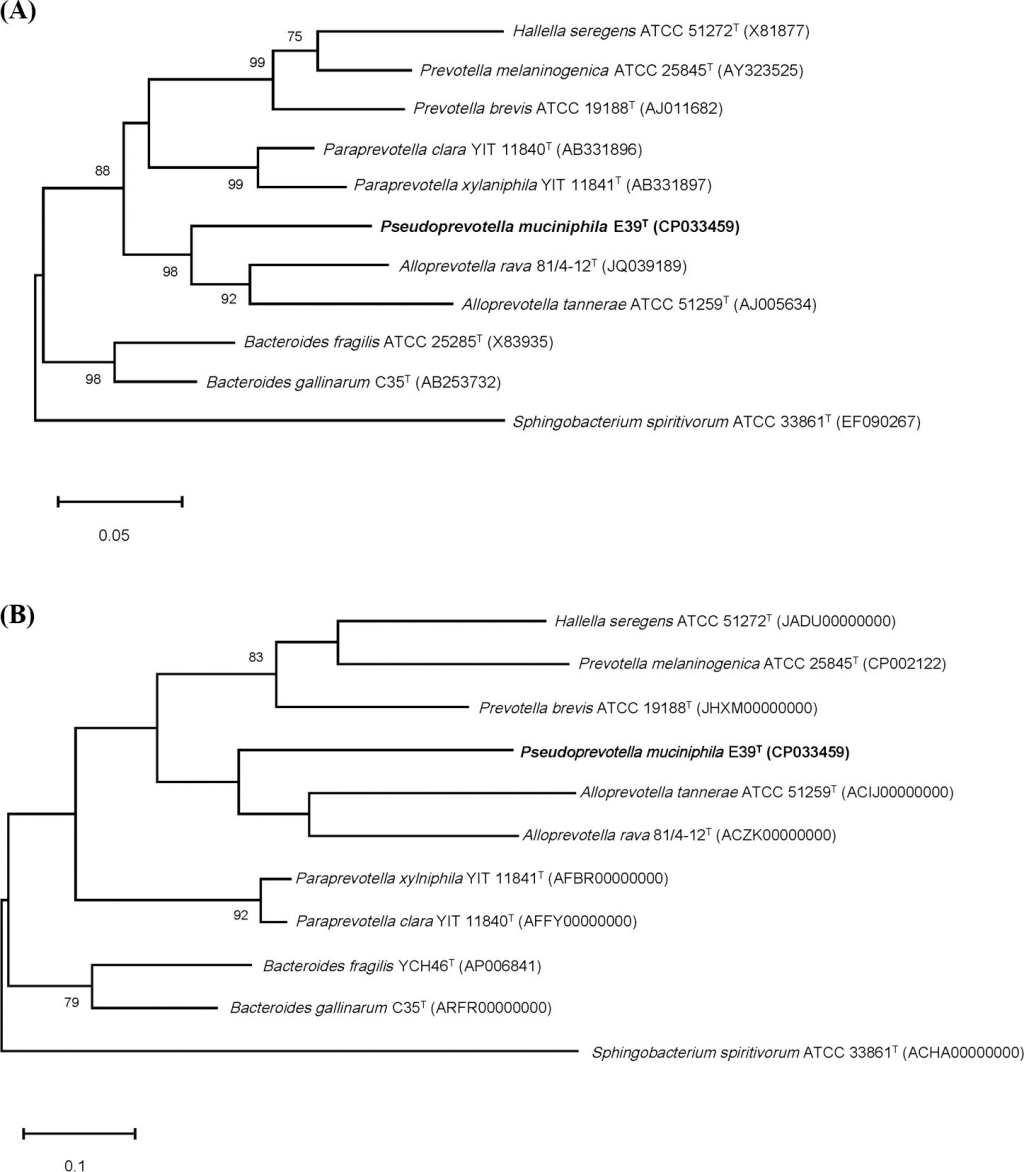
Phylogenetic relationship between strain E39^T^ and closely related strains within the order *Bacteroidales*, based on 16S rRNA gene sequences (A) and 92 bacterial core genes of the genomes (B). (A) The 16S rRNA-based tree was constructed with the maximum-likelihood method. Bootstrap values are shown on nodes in percentages and are based on 2,000 replicates. Only the bootstrap values over 70% are shown. The scale bar equals 0.05 changes per nucleotide position. (B) Bacterial core genes-based tree was constructed with the UBCG (up-to-date bacterial core gene) tool. The scale bar equals 0.1 changes per nucleotide position. The 16S rRNA gene (GenBank accession no., EF090267) and genome (ACHA00000000) sequences of *Sphingobacterium spiritivorum* ATCC 33861^T^ were used as an outgroup in the 16S rRNA gene- and bacterial core genes-based trees, respectively.

Comparative analysis of the 16S rRNA gene sequences revealed that strain E39^T^ was closely related to the genera *Alloprevotella*, *Paraprevotella*, *Prevotella*, and *Bacteroides*. *Alloprevotella rava* 81/4-12^T^, *Paraprevotella clara* YIT 11840^T^, *Paraprevotella xylaniphila* YIT 11841^T^, and *Bacteroides gallinarum* JCM 13658^T^ were most closely related to strain E39^T^ with 87.3%, 86.6%, 86.3%, and 85.9% 16S rRNA gene sequence similarities, respectively. The phylogenetic trees based on the ML algorithm and 92 bacterial core genes showed that strain E39^T^ was affiliated with the family *Prevotellaceae* and close to the genera *Alloprevotella* and *Paraprevotella* (Fig 1) [35–37]. Similarly, the phylogenetic trees based on the NJ and MP algorithms also showed that strain E39^T^ was affiliated with the family *Prevotellaceae* (S1 and S2 Figs). However, all of the phylogenetic trees showed that strain E39^T^ formed a distinct phylogenic lineage from the other genera. These molecular and phylogenetic analyses suggest that strain E39^T^ represents a novel genus of the family *Prevotellaceae*.

For additional conformation, ANI and dDDH were done with the genomes of strain E39^T^ and 4 reference strains. As a result, strain E39^T^ had lower ANI and dDDH values with *A*. *tannerae* (67.4%; 12.7%), *A*. *rava* (68.3%; 12.8%), *P*. *clara* (67.4%; 12.6%), and *P*. *melaninogenica* (66.7%; 12.7%) than single species thresholds (95–96% and 70%, respectively) [38].

### Phenotypic and chemotaxonomic characterization

**Fig 2:**
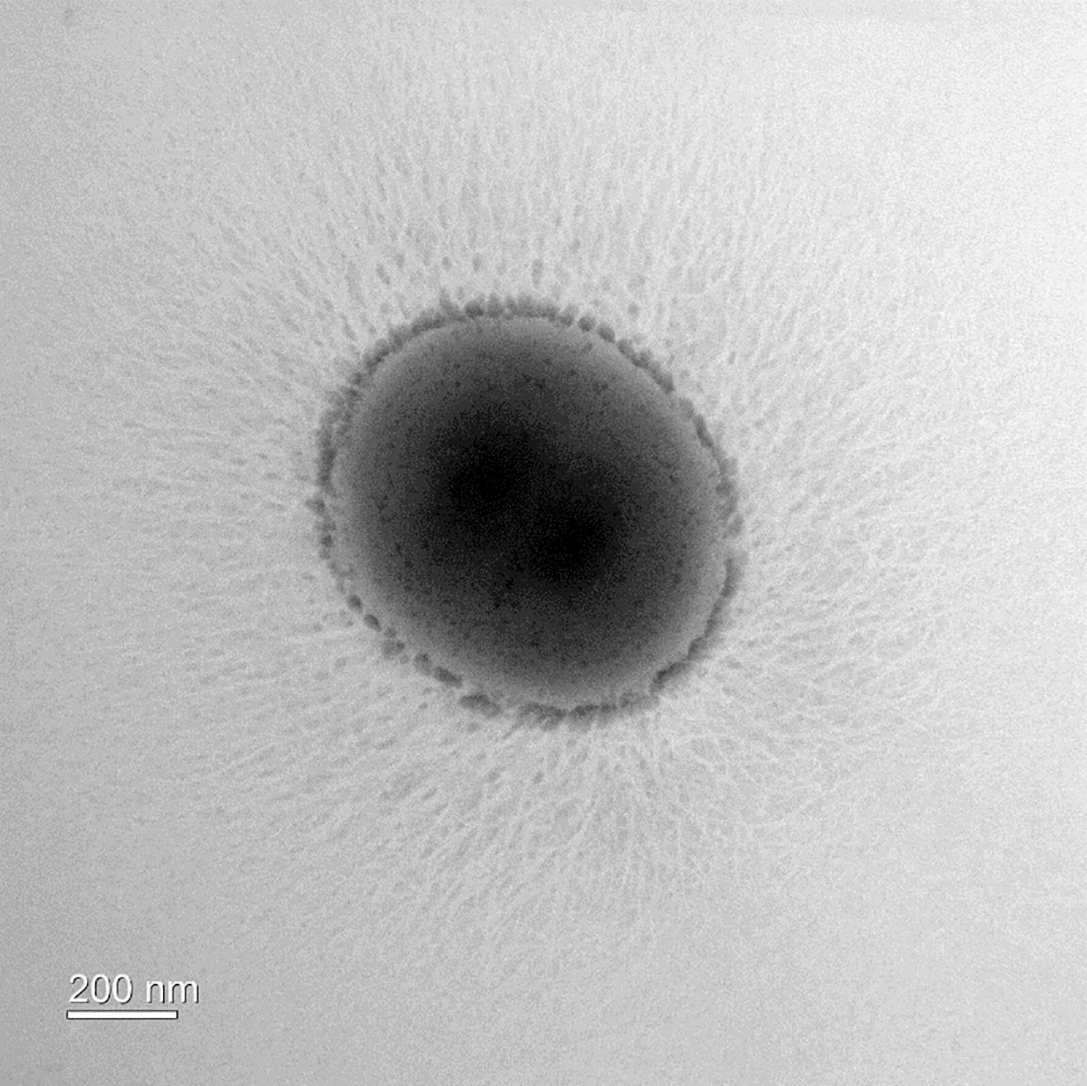
A transmission electron micrograph showing the general morphology of negatively stained cells of strain E39^T^. Cells were grown on basal mucin agar medium at 39°C for 3 days. Bar, 200 nm. Filamentous structures were observed from the cell surface.

The transmission electron microscopic analyses showed that the cells of strain E39^T^ were coccus in morphology (680–820 nm in diameter), and lacking in flagella (Fig 2). In addition, filamentous structures were observed from the cell surface. Among the various types of media, including BHI broth, TSB, Columbia broth, and anaerobe basal broth, strain E39^T^ could only grow on basal mucin medium. Cells grew at temperatures between 30 and 45°C, pH between 6.5 and 8.5, and in NaCl concentration between 0.0 and 1.0%. When the headspaces were filled with anaerobic gas, growth was observed in the absence of a reducing agent, but at a lower rate than in its presence. Cells did not grow in an aerobic atmosphere regardless of whether a reducing agent was present or not. These results demonstrate that strain E39^T^ prefers obligate anaerobic conditions but tolerates a trace amount of oxygen (S3 Fig).

In the API Rapid ID 32A panel, strain E39^T^ had positive activities of mucin-degrading enzymes including *β*-galactosidase, *N*-acetyl-*β*-glucosaminidase, and *α*-fucosidase [8]. Lack of urease activity suggest that strain E39^T^ may not attend to the digestion of urea, which is one of the roles of epimural bacteria. In particular, strain E39^T^ was distinguished from the reference taxa by a positive activity of arginine dihydrolase (Table 1). The major cellular fatty acids (> 5% of the total fatty acids) of strain E39^T^ were C_16:0_, C_18:0_, C_18:1_ *ω*9*c*, iso-C_15:0_, and anteiso-C_15:0_. Among strain E39^T^ and the reference taxa of the family *Prevotellaceae*, iso-C_15:0_ and anteiso-C_15:0_ were commonly detected as major cellular fatty acids. Especially, strain E39^T^ had higher portion of C_16:0_ and C_18:0_ compared with the reference taxa (Table 2). The major polar lipids of strain E39^T^ were phosphatidylethanolamine (PE), unidentified aminophospholipid (APL), and three unidentified lipids (L) (S4 Fig). Strain E39^T^ and the reference taxa of the family *Prevotellaceae* commonly contained PE and APL (except *P*. *melaninogenica*) (Table 1). Menaquinone (MK)-8 and MK-9 were identified from strain E39^T^ as major respiratory quinones. However, MK-9 was identified from *A*. *tanneare* and *A*. *rava*, the most closely related strains, as the sole respiratory quinones, while MK-10 and 11 and MK-7 and 11 were identified from *P*. *clara* and *P*. *melaninogenica*, respectively (Table 1). It has been reported that members of the family *Prevotellaceae* contain MK of between 10 to 13 as isoprenoid numbers [37]. However, MK-9 was identified from strain E39^T^ and *Alloprevotella* members and MK-8 was identified from only strain E39^T^ (Table 1), which differentiated strain E39^T^ from other genera of the family *Prevotellaceae*.

**Table 1:** Comparison of phenotype characteristics of strain E39^T^ and closely related taxa.

Characteristic	1	2	3	4	5
Morphology	Coccus	Rod	Rod	Rod	Rod
Optimal temperature (°C)	39	ND	35	37	37
Optimal pH	7.5	ND	7.0	ND	ND
Enzyme activity (API Rapid ID 32a) * of					
Arginine arylamidase	–	–	–	–	+
*β*-glucuronidase	–	–	–	w	–
*α*-arabinosidase	–	–	–	+	–
*α*-glucosidase	–	+	–	–	+
Mannose fermentation	–	–	+	+	+
Raffinose fermentation	–	–	+	+	+
*β*-galactosidase 6 phosphate	w	–	w	–	+
Arginine dihydrolase	+	–	–	–	–
*α*-galactosidase	+	–	w	–	–
*N*-acetyl-*β*-glucosaminidase	+	–	+	–	+
*α*-fucosidases	+	+	–	–	+
Leucyl glycine arylamidase	+	+	–	–	+
Glutamyl glutamic acid arylamidase	+	+	–	–	+
Glutamic acid decarboxylase	+	+	–	w	w
Polar lipids†*	PE, APL, L	PE, APL	PE, APL, PL, L	PE, APL, L	PE, L
Major quinones*	MK-8, MK- 9	MK-9	MK-9	MK-10, MK-11	MK-7, MK-11
DNA G+C content (mol%)‡	46.4	46.6	45.5	48.2	40.9

*Data were obtained from this study.

†PE, phosphatidylethanolamine; APL, unidentified aminophospholipid; PL, unidentified phospholipid; L, unidentified lipid.

‡The DNA G+C contents were calculated from the genome sequence.

Taxa: 1, strain E39^T^ [this study]; 2, *Alloprevotella tannerae* ATCC 51259^T^ [36]; 3, *Alloprevotella rava* 81/4-12^T^ [35]; 4, *Paraprevotella clara* JCM 14859^T^ [37]; 5, *Prevotella melaninogenica* ATCC 25845^T^ [45]. All strains are positive for the following characteristics: activity* of *β*-galactosidase, alkaline phosphatase and alanine arylamidase. All strains are negative for the following characteristics: activity* of urease, *β*-glucosidase, proline arylamidase, phenylalanine arylamidase, leucine arylamidase, pyroglutamic acid arylamidase, tyrosine arylamidase, glycine arylamidase, histidine arylamidase and serine arylamidase, reduction of nitrates, and indole production. Symbols: +, positive;–, negative; w, weak; ND, not determined.

**Table 2:** Cellular fatty acid compositions (%) of strain E39^T^ and closely related taxa.

Fatty acid	1	2	3	4	5
Saturated:					
C_12:0_	0.9	tr	tr	tr	tr
C_13:0_	0.9	–	–	–	–
C_14:0_	4.5	3.0	3.2	**11.6**	1.9
C_15:0_	–	–	–	–	–
C_16:0_	**24.9**	4.9	**14.5**	9.0	**8.7**
C_17:0_	1.2	tr	1.7	–	tr
C_18:0_	**18.3**	1.3	**6.7**	0.8	0.9
C_20:0_	0.8	tr	0.6	tr	tr
Unsaturated:					
C_13:1_ at 12–13	0.6	tr	2.2	1.5	2.2
C_18:1_ *ω*9c	**10.3**	0.9	**6.7**	**23.2**	**23.1**
Hydroxy:					
C_15:0_ 3-OH	0.9	tr	tr	tr	tr
C_16:0_ 3-OH	3.7	2.0	0.8	8.0	3.5
C_17:0_ 2-OH	tr	tr	0.7	tr	tr
iso-C_17:0_ 3-OH	2.1	**9.4**	**13.3**	0.6	3.6
Branched:					
iso-C_13:0_	1.6	3.5	1.0	tr	0.8
iso-C_14:0_	–	**8.8**	–	1.5	**12.2**
iso-C_15:0_	**9.0**	**26.6**	**8.4**	**11.0**	**5.6**
iso-C_16:0_	0.5	2.2	2.4	tr	1.0
iso-C_17:0_	3.0	4.2	**6.7**	tr	1.4
iso-C_18:0_	–	tr	1.0	–	tr
iso-C_19:0_	–	tr	1.1	–	0.8
anteiso-C_13:0_	tr	0.8	tr	tr	tr
anteiso-C_15:0_	**5.4**	**25.2**	**15.5**	**21.1**	**21.7**
anteiso-C_17:0_	0.9	1.9	2.4	tr	1.4
Summed feature*:					
3	0.9	tr	1.0	3.1	3.1
5	2.7	tr	–	–	–
8	3.5	tr	2.6	3.2	3.4

*Summed features represent groups of two fatty acids that cannot be separated by GLC with the MIDI system.

Summed feature 3, C_16:1_ *ω*7c and/or C_16:1_ *ω*6c; summed feature 5, C_18:0_ ante and/or C_18:2_ *ω*6,9c; summed feature 8, C_18:1_ *ω*7c and/or C_18:1_ *ω*6c.

Taxa: 1, strain E39^T^; 2, *Alloprevotella tannerae* ATCC 51259^T^; 3, *Alloprevotella rava* 81/4-12^T^; 4, *Paraprevotella clara* YIT 11840^T^; 5, *Prevotella melaninogenica* ATCC 25845^T^. All data were obtained from this study. Data are expressed as percentages of total fatty acids. In all strains, fatty acids that account for less than 0.5% are not shown. Major components (> 5.0%) are in bold. tr, trace amount (< 0.5%);–, not detected.

In conclusion, 16S rRNA gene and genome based analysis clearly supported identification of strain E39^T^ as a novel genus of the family *Prevotellaceae*. On top of that, strain E39^T^ was distinguished from the reference taxa by several traits, including coccus shape, growth only on media containing mucin, a positive activity for arginine dihydrolase, high portion of saturated fatty acids (C_16:0_ and C_18:0_),andtypical polar lipids (unidentified lipids), and MK-8 and MK-9 as major respiratory quinones (Table 3). Taken together, strain E39^T^ is considered to represent a new genus within the family *Prevotellaceae*, for which the name *Pseudoprevotella muciniphila* gen. nov., sp. nov. is proposed.

**Table 3:** Comparison of characteristics between *Pseudoprevotella* gen. nov. and closely related genera within the family *Prevotellaceae*.

Characteristic	1	2	3	4
Habitat	Rumen epithelium	Human oral cavity	Gut intestinal tract of mammals	Rumen, gut intestinal tract, and vagina of mammals
Metabolic end products from glucose	Acetate, succinate*†	Acetate, succinate	Acetate, succinate	Acetate, succinate
Major cellular fatty acids	C_16:0_, C_18:0_*	anteiso-C_15:0_, iso-C_15:0_*	anteiso-C_15:0_, iso-C_15:0_, C_18:1_ *ω*9c	anteiso-C_15:0_
Major quinones	MK-8, MK-9*	MK-9*	MK-10, MK-11	MK-10~MK-13
DNA G+C content (mol%)	46.4	45–47	48–49	40–52

*Data were obtained from this study.

† Metabolic end-products from mucin or mucin plus glucose.

Genera: 1, *Pseudoprevotella* gen. nov.; 2, *Alloprevotella* [35]; 3, *Paraprevotella* [37]; 4, *Prevotella* [45].

### Metabolite changes during mucin and glucose fermentation

**Fig 3:**
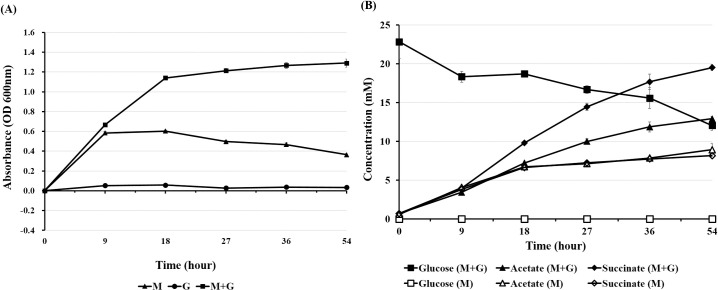
Growth of strain E39^T^ in the basal mucin (M), glucose (G), and mucin-glucose (M+G) media (A) and profiles of glucose, acetate, and succinate in the basal (M) and mucin-glucose (M+G) media during fermentation (B). Data are presented as mean ± standard error from triplicates.

The growth of strain E39^T^ was observed in the basal mucin medium and mucin-glucose medium, but not in glucose medium (Fig 3A). Strain E39^T^ grew in the mucin-glucose medium better than in the basal mucin medium. Metabolites including free sugars, organic acids, and amino acids were analyzed by ^1^H NMR spectroscopy. The concentration of acetate and succinate increased continuously in the both media (Fig 3B). The concentration of mannose and *N*-acetylglucosamine which are sugar residues of mucin structure, increased in both media during the early fermentation (0–9 h), and then decreased after 9 h (S5 Fig). Alanine, glycine, and valine were detected as the major amino acids during the fermentation in the both media (S6 Fig). Strain E39^T^ was not able to grow in media without mucin, and the growth of strain E39^T^ was promoted by the addition of glucose. These results suggest that mucin is an essential growth nutrient for strain E39^T^ and it can utilize glucose as an energy source only under the presence of mucin. Furthermore, it was shown that strain E39^T^ might utilize mucin as an energy source during the early fermentation and produce acetate, succinate, and several amino acids as major fermented end-products.

### Genomic features of the complete genome of *Pseudoprevotella muciniphila* E39^T^

Annotation analysis revealed that the genome of strain E39^T^ comprised 2,920,169 bases, 2,458 genes, 2,396 coding sequences (CDSs), 52 tRNA, 1 transfer-messenger RNA (tmRNA), and 9 rRNA (Table 4).

**Table 4:** Genomic features of *Pseudoprevotella muciniphila* E39^T^.

Number of Contigs	1		
Genome Size (bp)	2,920,169		
G+C Content (mol%)	46.4		
Number of Genes	2,458	Protein encoding genes	2,396
		tRNA	52
		tmRNA	1
		rRNA	9
Proteins with Predicted Functions	1,161		
Hypothetical or Uncharacterized Proteins	1,235		
Proteins with KEGG Annotations	971		
Carbohydrate-Active Enzymes with CAZyme Annotations	128	Glycoside Hydrolase	64
		Carbohydrate-Binding Module	5
		Glycosyl Transferase	49
		Polysaccharide Lyase	0
		Carbohydrate Esterase	10
Virulence Factors	17		
Antibiotic Resistant Genes	4		

#### (1) Mucin degradation and utilization

**Fig 4:**
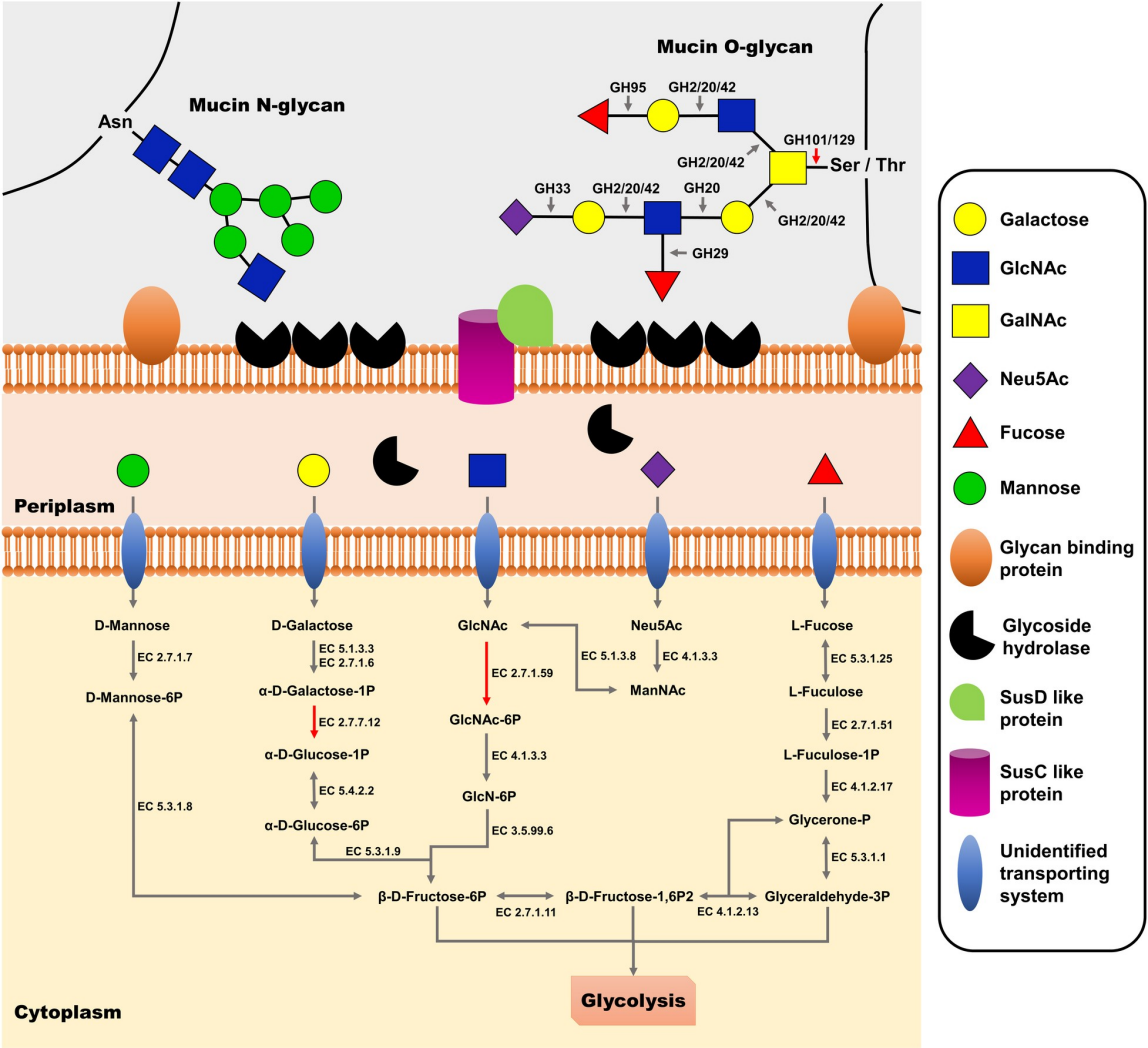
Proposed mucin degrading and utilizing pathways of the *P*. *muciniphila* E39^T^. These putative pathways were constructed based on CAZyme annotation, KEGG pathway analysis and BlastP analysis. Mucin is initially degraded into oligo- or monosaccharides by mucin degrading glycoside hydrolases (GHs) followed by transportation into periplasm by Sus-like outer membrane proteins. Additional degradation is occurred by periplasmic glycoside hydrolases and mucin-derived monosaccharides are imported through unidentified transporters and utilized as carbon sources. Metabolic pathways that are present in the *P*. *muciniphila* E39^T^ are depicted in gray, and metabolic pathways that are not present in the *P*. *muciniphila* E39^T^ are depicted in red. GlcNAc: *N*-acetlyglucosamine, GalNAc: *N*-acetylgalactosamine, Neu5Ac: sialic acid, ManNAc: *N*-acetylmannosamine.

Mucin is host-derived glycoprotein composed of a protein backbone, lots of O-glycan, and a small number of N-glycan. *N*-acetlygalactosamine (GalNAc) is O-glycosylated to proline-threonine-serine (PTS) domain of a protein backbone and addition of galactose or *N*-acetlyglucosamine (GlcNAc) forms 8 types of O-glycan core structures. Addition of extended core (galactose and GlcNAc) and terminal residues (sialic acid and fucose) makes mucin structure more complex [39]. To utilize mucin as an energy source, bacteria need to have series of enzymes to degrade complex mucin structure. Several glycoside hydrolases (GHs) were known as enzymes involved in mucin degradation such as sialidases (GH33), *α*-fucosidases (GH29, GH95), exo- and endo-*β*-*N*-acetylglucosaminidases (GH84, GH85), *β*-galactosidases (GH2, GH20, GH42), *α*-*N*-acetylglucosaminidases (GH89), endo-*β*1,4-galactosidases (GH98), and *α*-*N*-acetylgalactosaminidases (GH101, GH129) [39, 40]. In this study, the result of CAZyme annotation using dbCAN2 tool showed that *P*. *muciniphila* E39^T^ had 64 GHs, 5 carbohyrate-binding modules, 49 glycosyltransferases, 10 carbohydrate esterases, and no polysaccharide lyases (Table 4, S1 Dataset). Among 64 GHs, 28 GHs were putative enzymes involved in mucin degradation (Table 5). Compared to a Gram-negative mucin-degrading bacterium, *B*. *thetaiotaomicron*, *P*. *muciniphila* E39^T^ had fewer but similar kinds of mucin-degrading GHs. *Bacteroides thetaiotaomicron* has Sus-like systems for utilization of mucin glycan [41]. Glycan binding proteins on the cell surface bind polysaccharide and GHs partially degrade glycan. And then, two outer membrane proteins, homologs of SusD and SusC, import oligosaccharide into periplasm. After transportation of glycan from extracellular place to periplasm, glycan is degraded into monosaccharides by GHs and transferred to cytoplasm through inner membrane transporters [40, 41]. We confirmed that *P*. *muciniphila* E39^T^ also had homologs of SusC and SusD through a BlastP search against the genome of *P*. *muciniphila* E39^T^ (S1 Dataset). Based on a KEGG pathway analysis and BlastP analysis, we constructed putative mucin degrading pathway of *P*. *muciniphila* E39^T^. There were genes associated with metabolism of carbon sources including galactose, sialic acid (Neu5Ac), fucose, GlcNAc, and mannose on the results of KEGG pathway analysis (Fig 4). However, the proposed metabolic pathway was incomplete because of the absence of two genes (galactose 1-phosphate uridyltransferase, EC 2.7.7.12; *N*-acetylglucosamine kinase, EC 2.7.1.59) involved in galactose and GlcNAc metabolisms, respectively. In addition, *P*. *muciniphila* E39^T^ was negative in the mannose fermentation activity despite it harbored a mannose metabolic pathway. Further studies on mucin degrading pathway like monosaccharide transporting systems are needed to explain and understand mucin metabolic features of the *P*. *muciniphila* E39^T^.

**Table 5:** List of predicted mucin degrading enzymes in the complete genome of strain E39^T^.

Mucin degrading enzymes	Glycoside hydrolases (GHs)	Locus ID^a^	KEGG ID	Prokka annotation
*β*-galactosidases	GH2	L_00290	K01190 (*β*-galactosidase)	*β*-galactosidase
		L_01043	K01190 (*β*-galactosidase)	*β*-galactosidase
		L_01673	K01190 (*β*-galactosidase)	Evolved *β*-galactosidase subunit *α*
	GH20	L_00323	K12373 (hexosaminidase)	*β*-hexosaminidase
		L_00864	–	*β*-*N*-acetylhexosaminidase
		L_00865	–	*β*-*N*-acetylhexosaminidase
		L_01112	K12373 (hexosaminidase)	*β*-hexosaminidase
		L_01187	K12373 (hexosaminidase)	*β*-hexosaminidase
		L_01443	K12373 (hexosaminidase)	*β*-hexosaminidase
		L_01556	K12373 (hexosaminidase)	*β*-hexosaminidase
		L_01824	K12373 (hexosaminidase)	*β*-hexosaminidase
	GH42	–	–	–
Endo-*β*1,4-galactosidases	GH98	–	–	–
*α*-*N*-acetylgalactosaminidases	GH101	–	–	–
	GH129	–	–	–
Exo- and endo-*β*-*N*-acetylglucosaminidases	GH84	L_00360	K01197 (hyaluronoglucosaminidase)	Hyaluronoglucosaminidase
		L_01917	K01197 (hyaluronoglucosaminidase)	O-GlcNAcase
	GH85	L_00833	–	Hypothetical protein
		L_01606	–	Hypothetical protein
*α*-*N*-acetylglucosaminidases	GH89	L_01212	K01205 (*α*-*N*-acetylglucosaminidase)	Hypothetical protein
		L_01965	K01205 (*α*-*N*-acetylglucosaminidase)	Hypothetical protein
Sialidases	GH33	L_00875	–	Hypothetical protein
		L_01672	K01186 (sialidase-1)	Sialidase
		L_02283	–	Sialidase
Fucosidases	GH29	L_00284	K01206 (*α*-L-fucosidase)	Hypothetical protein
		L_00599	K01206 (*α*-L-fucosidase)	Hypothetical protein
		L_00887	K01206 (*α*-L-fucosidase)	Hypothetical protein
		L_00979	K01206 (*α*-L-fucosidase)	Hypothetical protein
		L_01701	K01206 (*α*-L-fucosidase)	Hypothetical protein
	GH95	L_00982	K15923 (*α*-L-fucosidase 2)	Hypothetical protein
		L_01250	K15923 (*α*-L-fucosidase 2)	Hypothetical protein
		L_02348	K15923 (*α*-L-fucosidase 2)	Hypothetical protein

^a^Locus IDs are the results of CAZyme annotation using dbCAN2 tool.

#### (2)Extracellular polymeric substances biosynthesis

Bacteria produce biofilms for various purposes. Biofilms are composed of extracellular polymeric substances including nucleic acids, lipids, proteins, and exopolysaccharides and this complex compounds have a wide range of roles in adhesion to other bacterial cells or host cells, protection from stresses such as antibiotic substances or harmful chemicals, and provision of structure for stratification against rapid environmental changes [42]. In the Wzx/Wzy-dependent pathway, one of exopolysaccharides biosynthesis pathways, glycosyltransferases assemble repeating units and a flippase (Wzx protein) translocates the units into the periplasmic place. The repeating units are elongated by a polymerase (Wzy protein) and transported across the outer membrane through a polysaccharide export protein [43]. *P*. *muciniphila* E39^T^ also produced branch-shaped extracellular structures (Fig 2) and these structures are predicted to contribute to adhesion to mucin or host cells. We identified that *P*. *muciniphila* E39^T^ had 1 putative extracellular polysaccharide biosynthesis locus (L_02166 –L_02195) through a BlastP search against the genome of *P*. *muciniphila* E39^T^ (S1 Dataset). There were 8 putative glycosyltransferases (L_02166, L_02169, L_02175, L_02179, L_02180, L_02181, L_02183, L_02184), 1 polymerase (L_02174), 1 flippase (L_02189), 1 serine acetyltransferase (L_02182), 1 N-acetyltransferase (L_02182), 1 aminotransferase (L_02188), 1 polysaccharide pyruvyl transferase (L_02191), and 1 polysaccharide export protein (L_02193).

#### (3)Virulence factors

Bacterial virulence factors allow bacteria to survive in the host, participating in adhesion, colonization, invasion, evasion or inhibition of immune responses, etc [44]. *P*. *muciniphila* E39^T^ had 17 putative virulence factors and they involved in adherence (*KpsF*, *htpB*, *glf*, *hasB*), antiphagocytosis (*cps4J*, *cps4K*, *cps4L*, *hasB*), immune evasion (*tviC*), stress reaction (*clpC*, *clpP*), O-antigen (*galE*, *fcl*), lipopolysaccharide (*gmd*), and metabolic adaptation (*panD*) (S1 Dataset). The virulence factors encoded in these genes may enable *P*. *muciniphila* E39^T^ to survive on the rumen wall, a place which host’s defense mechanism is most active in the rumen, by attaching to epithelial cells and avoiding the host’s immune responses. The absence of genes encoding exotoxin and involved in invasion suggests that *P*. *muciniphila* E39^T^ may have low pathogenicity. However, we cannot ignore the possibility that *P*. *muciniphila* E39^T^ is a potential pathogen because of its endotoxin (lipopolysaccharide) and mucinolytic ability. Further researches at molecular level or *in vivo* studies are required to determine the pathogenicity of *P*. *muciniphila* E39^T^.

## Conclusions

The genetic, physiological, and chemotaxonomic features support that strain E39^T^ represents a novel genus of the family *Prevotellaceae*. As such, the name *Pseudoprevotella muciniphila* gen. nov., sp. nov. is proposed. *Pseudoprevotella muciniphila* E39^T^ was isolated from the bovine rumen epithelium and this bacterium utilized mucin as a sole carbon source. The functional annotation of the complete genome of *P*. *muciniphila* E39^T^ supported that *P*. *muciniphila* E39^T^ possess a series of mucin degrading enzymes and putative mucin-degrading pathway. In addition, *P*. *muciniphila* E39^T^ is predicted to have putative metabolisms to synthesize extracellular polymeric substances and virulence factors for adhering to rumen epithelial cells and evading the host’s immune responses. In short, this study contributes to discovery of a novel mucin-degrading bacterium which has a potential ability to significantly affect host’s physiology and its putative metabolic pathways which can assist to predict its function in epimural community.

### Description of *Pseudoprevotella muciniphila* gen. nov., sp. nov.

*Pseudoprevotella* (Pseu.do.pre.vo.tel′la. Gr. adj. *pseudês* false; N.L. n. fem. n. *Prevotella* a bacterial generic name; N.L. fem. n. *Pseudoprevotella* false *Prevotella*).

The cells are strictly anaerobic, non-motile, Gram-negative and coccus in shape. The major cellular fatty acids are C_16:0_, C_18:0_, C_18:1_ *ω*9*c*, iso-C_15:0_, and anteiso-C_15:0_. The main polar lipids are phosphatidylethanolamine (PE), unidentified aminophospholipid (APL), and three unidentified lipids. The major respiratory quinones are MK-8 and MK-9. The major fermented end-products of mucin are acetate and succinate. The genus is a member of the family *Prevotellaceae* of the phylum *Bacteroidetes*. The type species is *Pseudoprevotella muciniphila*.

### Description of *Pseudoprevotella muciniphila* sp. nov.

*Pseudoprevotella muciniphila* (mu.ci.ni′phi.la. N.L. neut. n. *mucinum* mucin; Gr. adj. *philos* loving; N.L. fem. adj. *muciniphila* mucin-loving).

In addition to the characteristics provided in the genus description above, this species grows at 30–45°C (optimum, 39°C), at pH 6.5–8.5 (optimum, pH 7.5), and in the presence of 0.0–1.0% (w/v) NaCl (optimum, 0.0–0.5%). This species had arginine dihydrolase, *α*-galactosidase, *β*-galactosidase, *β*-galactosidase-6-phosphate, *N*-acetyl-*β*-glucosaminidase, glutamic acid decarboxylase, *α*-fucosidase, alkaline phosphatase, leucyl glycine arylamidase, alanine arylamidase, and glutamyl glutamic acid arylamidase activity, but lacked urease, *α*-glucosidase, *β*-glucosidase, *α*-arabinosidase, *β*-glucuronidase, mannose fermentation, raffinose fermentation, reduction of nitrates, indole production, arginine arylamidase, proline arylamidase, phenylalanine arylamidase, leucine arylamidase, pyroglutamic acid arylamidase, tyrosine arylamidase, glycine arylamidase, histidine arylamidase, and serine arylamidase activity. This species has a DNA G+C content of 46.4 mol%. The type strain is E39^T^ (KCTC 15717^T^ = JCM 32621^T^), and it was isolated from the rumen epithelium of Korean cattle.

## Supporting information

S1 Fig Neighbor-joining tree showing the phylogenetic relationship between strain E39^T^ and closely related strains within the order *Bacteroidales*, based on 16S rRNA gene sequences. Bootstrap values over 70% are shown on the nodes as percentages of 2,000 replicates. *Sphingobacterium spiritivorum* ATCC 33861^T^ (EF090267) was used as an outgroup. Bar indicates 0.02 changes per nucleotide position.
(DOCX)

S2 Fig Maximum-parsimony tree showing the phylogenetic relationship between strain E39^T^ and closely related strains within the order *Bacteroidales*, based on 16S rRNA gene sequences. Bootstrap values over 70% are shown on the nodes as percentages of 2,000 replicates. *Sphingobacterium spiritivorum* ATCC 33861^T^ (EF090267) was used as an outgroup. Bar indicates 50 changes per nucleotide position.
(DOCX)

S3 Fig Growth of strain E39^T^ in the absence of a reducing agent or in the aerobic condition. NROX: No reducing agent and aerobic headspace, NRAN: No reducing agent and anaerobic headspace, PROX: presence of reducing agent and aerobic headspace, PRAN: presence of reducing agent and anaerobic headspace. Data are presented as mean ± standard error from triplicates.
(DOCX)

S4 Fig Total polar lipid profiles of strain E39^T^ and closely related strains within the family *Prevotellaceae*. Solvent systems: (I) chloroform-methanol-water (65:25:4, v/v/v); (II) chloroform-acetic acid-methanol-water (80:15:12:4, v/v/v/v). The TLC plates were sprayed with 10% ethanolic molybdatophosphoric acid. (A) strain E39^T^, (B) *Alloprevotella tannerae*, (C) *Alloprevotella rava*, (D) *Paraprevotella clara*, (E) *Prevotella melaninogenica*. PE, phosphatidylethanolamine; APL, unidentified aminophospholipids; PL, unidentified phospholipids; L, unidentified polar lipids.
(DOCX)

S5 Fig Profiles of galactose, *N*-acetylglucosamine, and mannose in the mucin-glucose (A) and basal mucin (B) media during fermentation. Data are presented as mean ± standard error from triplicates.
(DOCX)

S6 Fig Profile of major amino acids in the mucin-glucose (A) and basal mucin (B) media during fermentation. Data are presented as mean ± standard error from triplicates.
(DOCX)

S1 Dataset Genome features of *Pseudoprevotella muciniphila* E39^T^. (XLSX)
