# Supplementary material for: Pseudoprevotella muciniphila gen. nov., sp. nov., a mucin-degrading bacterium attached to the bovine rumen epithelium
Source: PLoS One. 2021 May 20;16(5):e0251791. doi: 10.1371/journal.pone.0251791 (PMC8136628; doi:10.1371/journal.pone.0251791)
Supplement: S2 Fig — Bootstrap values over 70% are shown on the nodes as percentages of 2,000 replicates. Sphingobacterium spiritivorum ATCC 33861T (EF090267) was used as an outgroup. Bar indicates 50 changes per nucleotide position. (DOCX) [file pone.0251791.s002.docx]

**S2 Fig. Maximum-parsimony tree showing the phylogenetic relationship between strain E39^T^ and closely related strains within the order *Bacteroidales*, based on 16S rRNA gene sequences.** Bootstrap values over 70% are shown on the nodes as percentages of 2,000 replicates. *Sphingobacterium spiritivorum* ATCC 33861^T^ (EF090267) was used as an outgroup. Bar indicates 50 changes per nucleotide position.

**

**
