# Supplementary material for: Pseudoprevotella muciniphila gen. nov., sp. nov., a mucin-degrading bacterium attached to the bovine rumen epithelium
Source: PLoS One. 2021 May 20;16(5):e0251791. doi: 10.1371/journal.pone.0251791 (PMC8136628; doi:10.1371/journal.pone.0251791)
Supplement: S3 Fig — NROX: No reducing agent and aerobic headspace, NRAN: No reducing agent and anaerobic headspace, PROX: presence of reducing agent and aerobic headspace, PRAN: presence of reducing agent and anaerobic headspace. Data are presented as mean ± standard error from triplicates. (DOCX) [file pone.0251791.s003.docx]

**S3 Fig. Growth of strain E39^T^ in the absence of a reducing agent or in the aerobic condition.** NROX: No reducing agent and aerobic headspace, NRAN: No reducing agent and anaerobic headspace, PROX: presence of reducing agent and aerobic headspace, PRAN: presence of reducing agent and anaerobic headspace. Data are presented as mean ± standard error from triplicates.

**
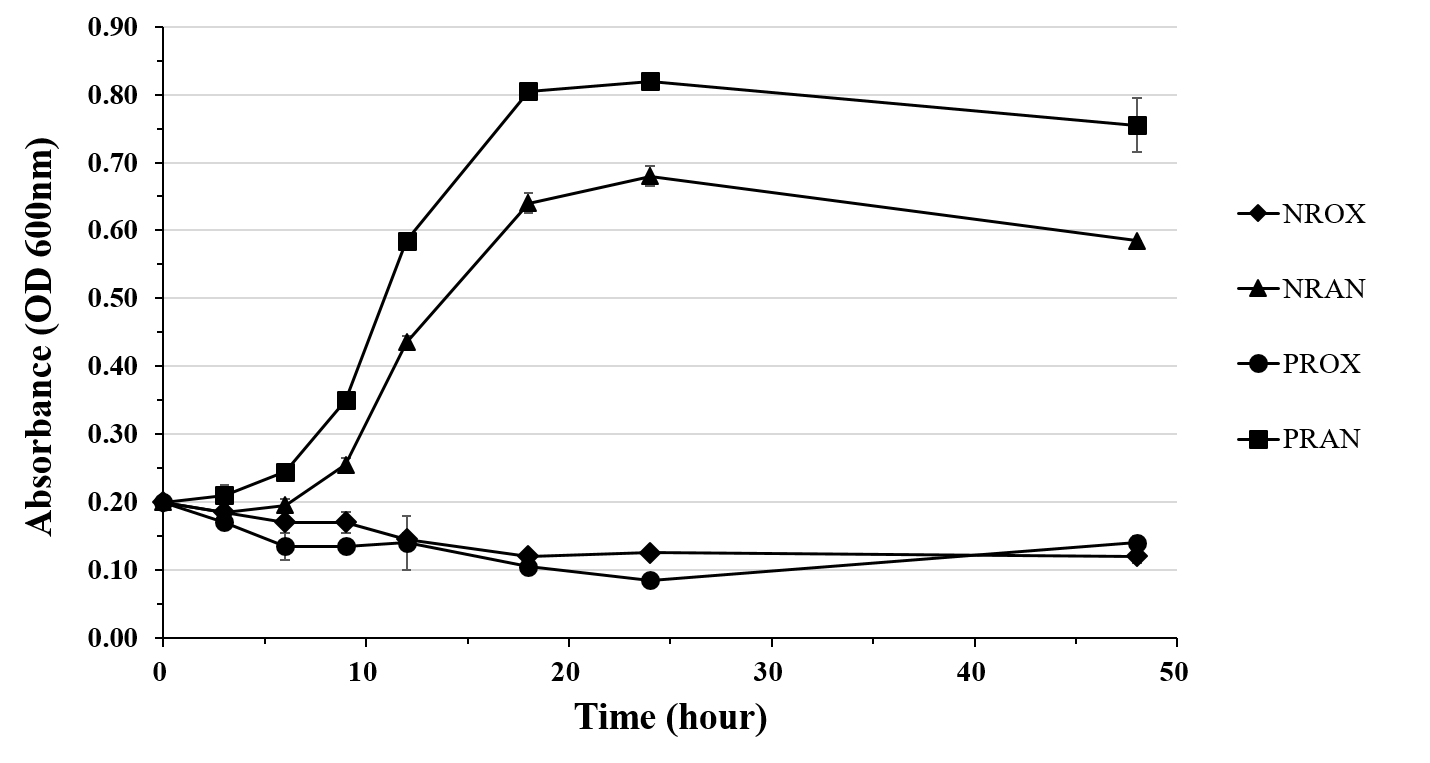
**
