# Supplementary material for: Pseudoprevotella muciniphila gen. nov., sp. nov., a mucin-degrading bacterium attached to the bovine rumen epithelium
Source: PLoS One. 2021 May 20;16(5):e0251791. doi: 10.1371/journal.pone.0251791 (PMC8136628; doi:10.1371/journal.pone.0251791)
Supplement: S4 Fig — Solvent systems: (I) chloroform-methanol-water (65:25:4, v/v/v); (II) chloroform-acetic acid-methanol-water (80:15:12:4, v/v/v/v). The TLC plates were sprayed with 10% ethanolic molybdatophosphoric acid. (A) strain E39T, (B) Alloprevotella tannerae, (C) Alloprevotella rava, (D) Paraprevotella clara, (E) Prevotella melaninogenica. PE, phosphatidylethanolamine; APL, unidentified aminophospholipids; PL, unidentified phospholipids; L, unidentified polar lipids. (DOCX) [file pone.0251791.s004.docx]

**S4 Fig. Total polar lipid profiles of strain E39^T^ and closely related strains within the family *Prevotellaceae*.** Solvent systems: (I) chloroform-methanol-water (65:25:4, v/v/v); (II) chloroform-acetic acid-methanol-water (80:15:12:4, v/v/v/v). The TLC plates were sprayed with 10 % ethanolic molybdatophosphoric acid. (A) strain E39^T^, (B) *Alloprevotella tannerae*, (C) *Alloprevotella rava*, (D) *Paraprevotella clara*, (E) *Prevotella melaninogenica*. PE, phosphatidylethanolamine; APL, unidentified aminophospholipids; PL, unidentified phospholipids; L, unidentified polar lipids.

**
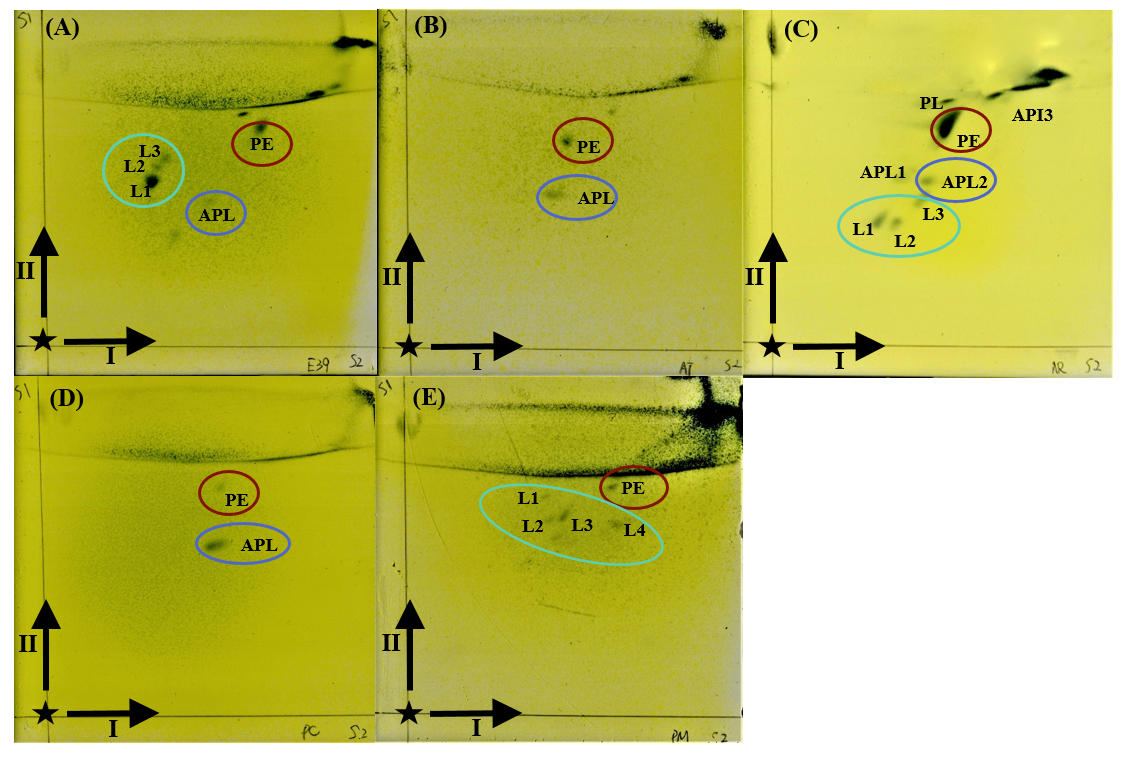
**
