# Supplementary material for: Pseudoprevotella muciniphila gen. nov., sp. nov., a mucin-degrading bacterium attached to the bovine rumen epithelium
Source: PLoS One. 2021 May 20;16(5):e0251791. doi: 10.1371/journal.pone.0251791 (PMC8136628; doi:10.1371/journal.pone.0251791)
Supplement: S5 Fig — Profiles of galactose, N-acetylglucosamine, and mannose in the mucin-glucose (A) and basal mucin (B) media during fermentation. Data are presented as mean ± standard error from triplicates. (DOCX) [file pone.0251791.s005.docx]

**S5 Fig. Profiles of lactate, galactose, *N*-acetylglucosamine, and mannose in the mucin-glucose (A) and basal mucin (B) media during fermentation.** Data are presented as mean ± standard error from triplicates.

**
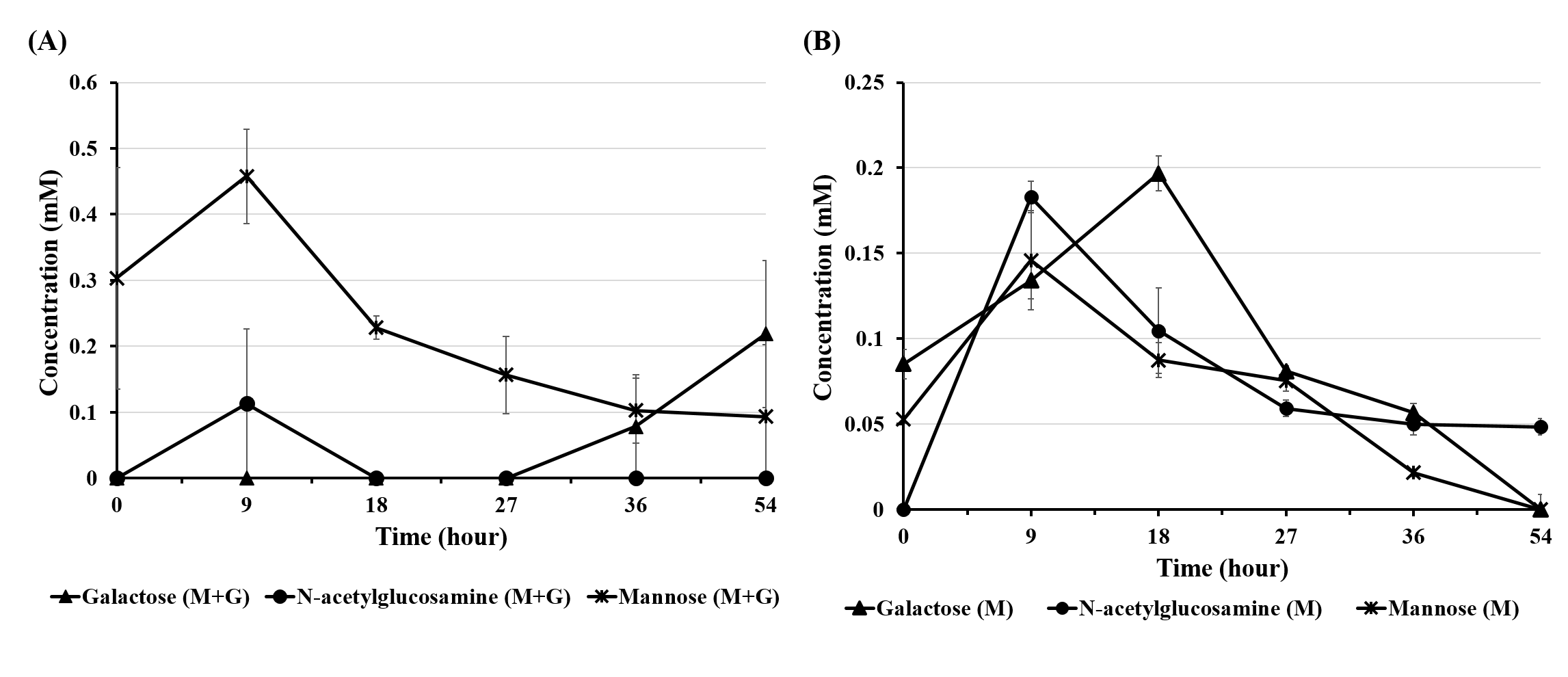
**
