# Supplementary material for: Pseudoprevotella muciniphila gen. nov., sp. nov., a mucin-degrading bacterium attached to the bovine rumen epithelium
Source: PLoS One. 2021 May 20;16(5):e0251791. doi: 10.1371/journal.pone.0251791 (PMC8136628; doi:10.1371/journal.pone.0251791)
Supplement: S6 Fig — Profile of major amino acids in the mucin-glucose (A) and basal mucin (B) media during fermentation. Data are presented as mean ± standard error from triplicates. (DOCX) [file pone.0251791.s006.docx]

**S6 Fig. Profile of major amino acids in the mucin-glucose (A) and basal mucin (B) media during fermentation.** Data are presented as mean ± standard error from triplicates.

**
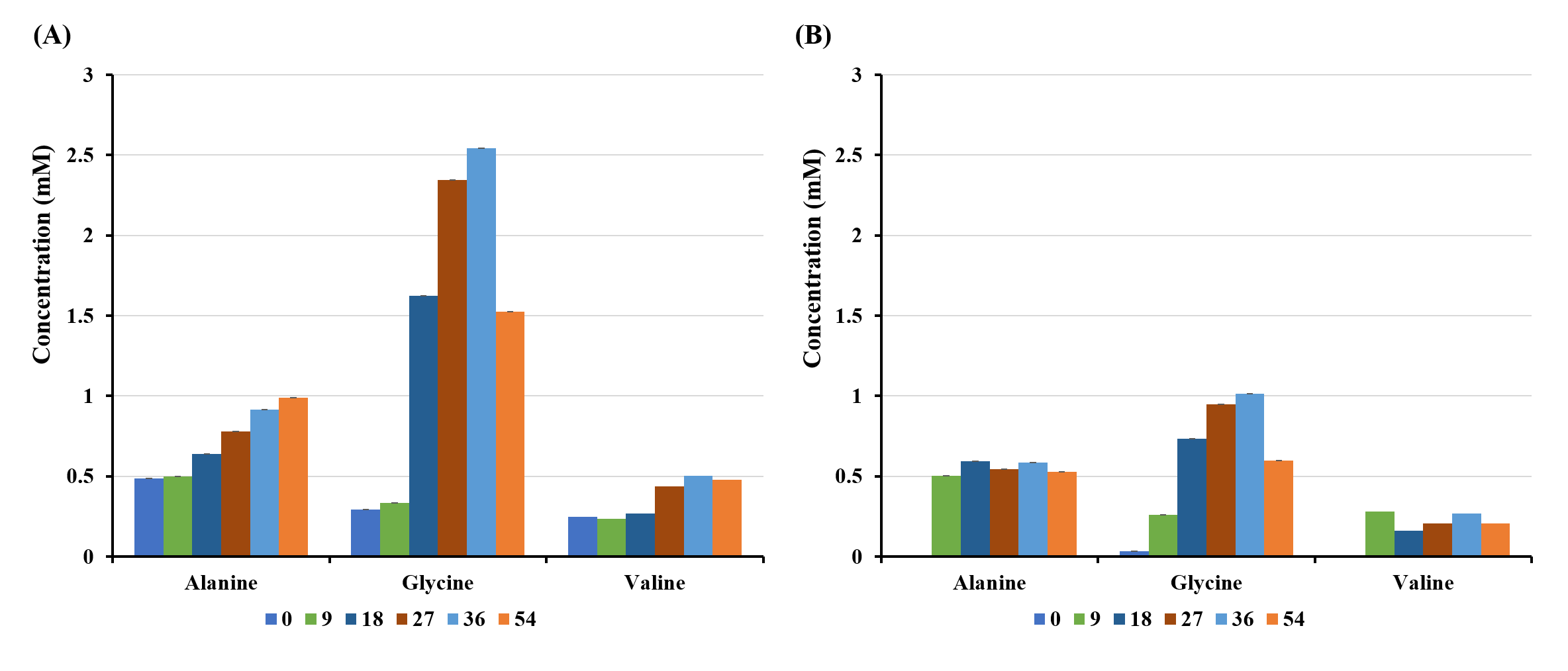
**
